# Detection, Properties, and Frequency of Local Calcium Release from the Sarcoplasmic Reticulum in Teleost Cardiomyocytes

**DOI:** 10.1371/journal.pone.0023708

**Published:** 2011-08-29

**Authors:** Anna Llach, Cristina E. Molina, Enrique Alvarez-Lacalle, Lluis Tort, Raul Benítez, Leif Hove-Madsen

**Affiliations:** 1 Cardiovascular Research Centre CSIC and IIB Sant Pau, Hospital de Sant Pau, Barcelona, Barcelona, Spain; 2 Departamento Biología Celular, Fisiología e Inmunología, Universitat Autònoma de Barcelona, Barcelona, Spain; 3 Departamento Ingeniería de Sistemas, Automática e Informática Industrial, Universitat Politècnica de Catalunya, Barcelona, Spain; University of Queensland, Australia

## Abstract

Calcium release from the sarcoplasmic reticulum (SR) plays a central role in the regulation of cardiac contraction and rhythm in mammals and humans but its role is controversial in teleosts. Since the zebrafish is an emerging model for studies of cardiovascular function and regeneration we here sought to determine if basic features of SR calcium release are phylogenetically conserved. Confocal calcium imaging was used to detect spontaneous calcium release (calcium sparks and waves) from the SR. Calcium sparks were detected in 16 of 38 trout atrial myocytes and 6 of 15 ventricular cells. The spark amplitude was 1.45±0.03 times the baseline fluorescence and the time to half maximal decay of sparks was 27±3 ms. Spark frequency was 0.88 sparks µm^−1^ min^−1^ while calcium waves were 8.5 times less frequent. Inhibition of SR calcium uptake reduced the calcium transient (F/F_0_) from 1.77±0.17 to 1.12±0.18 (p = 0.002) and abolished calcium sparks and waves. Moreover, elevation of extracellular calcium from 2 to 10 mM promoted early and delayed afterdepolarizations (from 0.6±0.3 min^−1^ to 8.1±2.0 min^−1^, p = 0.001), demonstrating the ability of SR calcium release to induce afterdepolarizations in the trout heart. Calcium sparks of similar width and duration were also observed in zebrafish ventricular myocytes. In conclusion, this is the first study to consistently report calcium sparks in teleosts and demonstrate that the basic features of calcium release through the ryanodine receptor are conserved, suggesting that teleost cardiac myocytes is a relevant model to study the functional impact of abnormal SR function.

## Introduction

Calcium release from the sarcoplasmic reticulum (SR) plays a central role in the regulation of the contraction and rhythm of the mammalian heart [1]. Spontaneous release of calcium from the SR through a small cluster of calcium release channels in the SR (ryanodine receptor; RyR2) gives rise to calcium sparks [2]. The calcium sparks are elementary events in the cardiac excitation coupling and a synchronized calcium release from a large number of ryanodine receptor clusters, induced by the action potential, activates contraction under normal circumstances [3]. Contraction can also be elicited by asynchronous calcium release from the SR, which typically occurs under pathological conditions in the mammalian and human heart [4], [5], [6]. Such asynchronous release gives rise to calcium waves, and subsequent membrane depolarizations [7] that can induce arrhythmias [8].

Because of this predominant role of the SR in the regulation of cardiac contraction and rhythm, calcium release from the SR through the ryanodine receptor might be expected to be a conserved mechanism, and investigation of the functional role of the SR in the teleost heart, which phylogenetically is the ancestor of other vertebrate hearts, may bring important information on evolutionary changes in the SR function.

Direct measurements of calcium release from the SR in the ectothermic vertebrate heart are however rather limited. Moreover, myocytes from the lower vertebrate heart are long and thin without T-tubules, suggesting that trans-sarcolemmal calcium entry is sufficient to activate contraction in this heart. Indeed, calcium entry through L-type calcium channels is considered the predominating source of calcium in the frog ventricle [9], [10], and experiments examining calcium release from the SR failed to detect calcium release from the SR in frog ventricle [11]. On the other hand, several studies on amphibian [12] and some teleost hearts [13], [14] have reported smaller L-type calcium current densities that can only account for 10–25% of the total calcium transient [1]. Therefore, additional calcium sources, higher calcium sensitivity of the myofilaments, and/or a different spatial arrangement of the contractile machinery are required for activation of contraction in these hearts. Indeed, Na^+^-Ca^2+^ exchange could contribute with an amount equal to that of the L-type calcium current [15], [16], [17], and myofilament calcium sensitivity is also higher in the teleost heart [18]. This data, combined with a peripheral localization of the myofilaments [19], [20], small effects of SR inhibition with ryanodine [21], and failure to detect calcium sparks in isolated trout cardiomyocytes in a previous study [22], has been taken as support for a dominant role of trans-sarcolemmal calcium entry in the activation of contraction in the teleost heart [20].

In contrast to this, ultrastructural studies have identified SR in the teleost heart, primarily underneath the sarcolemma [19], [20]. Furthermore, the SR is capable of accumulating and releasing significant amounts of calcium in trout atrial and ventricular myocytes [15], [23], [24], [25], suggesting that the SR may participate actively in the E-C coupling. However, the presence of calcium sparks, the elementary event underlying synchronized and spontaneous calcium release from the SR, has not been established in the ectothermic vertebrate heart. Therefore, the aim of the present study was to test the hypothesis that calcium sparks are present in teleost cardiomyocytes and that the spark properties are conserved. This was achieved by recording calcium sparks in cardiac myocytes from two teleost species, the rainbow trout and the zebrafish.

Our results reveal the presence of calcium sparks in isolated atrial and ventricular myocytes from the trout heart. Both the physical properties and the frequency of calcium sparks were similar to those reported in mammals, suggesting that the key features of calcium release through the ryanodine receptor are conserved from teleost to mammal. This, combined with the presence of early and delayed afterdepolarizations in trout cardiomyocytes suggests that the SR plays an important role in excitation-contraction coupling in the teleost heart and provides novel support for teleost cardiomyocytes as a relevant model for studies of the impact of genetic or pharmacological manipulation of SR function on cardiac arrhythmogenesis.

## Methods

### Cell isolation and patch-clamp technique

Trout atrial and ventricular myocytes were obtained by enzymatic digestion of the heart and subjected to patch-clamp technique using a HEKA EPC-10 amplifier and experimental protocols and conditions described previously [13], [23]. Action potentials and afterdepolarizations were recorded using the current-clamp technique and experimental protocols and conditions previously described [26]. Spontaneous membrane depolarizations were only considered afterdepolarizations if they were associated with a concurrent cell shortening. Similarly, transient inward currents (I_TI_) resulting from spontaneous calcium waves were only considered if they were associated with a concurrent cell shortening. Zebrafish ventricular myocytes were obtained by enzymatic perfusion of the heart as recently described [27].

### Ethic Statement

The myocte isolation procedure was approved by the local ethical committee at the Universitat Autònoma de Barcelona (DARP no. 1017), and they were conducted in accordance with the Guide for the Care and Use of Laboratory Animals published by the US National Institutes of Health.

### Confocal calcium imaging

Ca^2+^-sparks, Ca^2+^-waves and electrically stimulated Ca^2+^-transients were detected using a laser scanning confocal microscope (Leica SP2 AOBS, Germany) and a 63× objective with a numerical aperture of 1.3. Cells were first incubated with 2.5 to 5 µM fluo-4 acetoxymethylester for 10–20 minutes at room temperature, then washed and left at rest for deesterification for at least 30 minutes before experiments were started. Changes in the intracellular [Ca^2+^] were detected using the line-scanning mode at a scan rate of 1 kHz. Cells were excited at 488 nm. In order to minimize bleaching, fluorescence emission was collected between 505 and 650 nm and the excitation beam was attenuated to 1–5%. Experiments were performed in a physiological saline containing (in mM): 132 NaCl, 2.5 KCl, 4 NaHCO_3_, 0.33 NaH_2_PO_4_, 2 CaCl_2_, 1.6 MgCl_2_, 10 HEPES, 5 glucose, and 5 pyruvic acid. pH was adjusted to 7.5 with NaOH, at 20°C.

**Figure 1:**
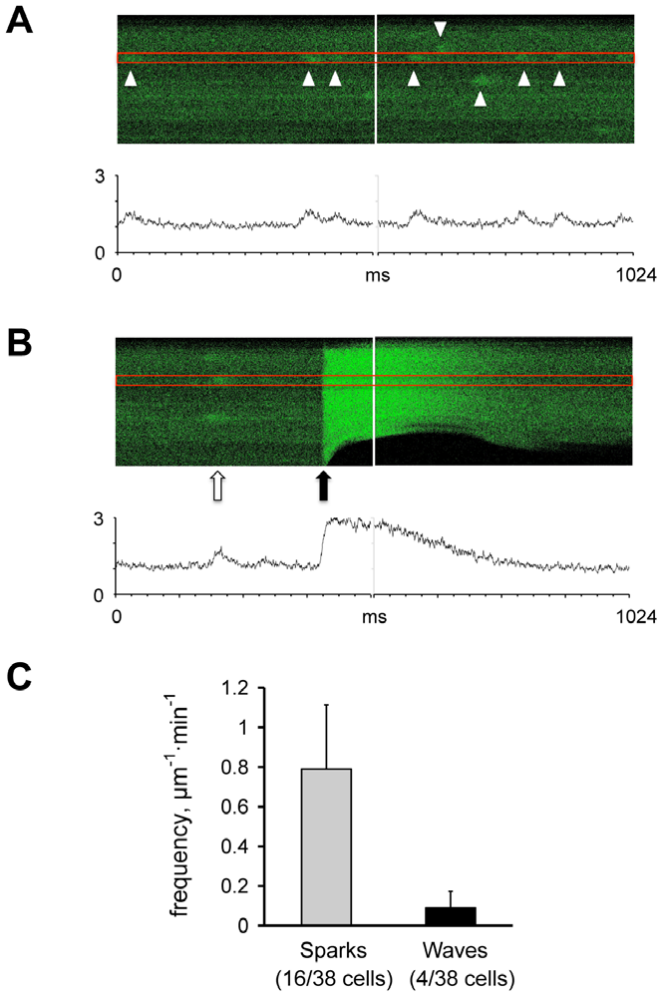
Calcium sparks are present in trout atrial myocytes. **A** The upper panel shows calcium sparks (white arrowheads) recorded in the line-scanning mode. The lower panel shows the fluorescence intensity (F/F_0_) recorded in a 3 µm wide band delimited by the two red lines. **B** Comparison of spontaneous calcium sparks (white arrow) and a field stimulated calcium transient (black arrow). The corresponding calcium transients recorded in a 3 µm wide band delimited by the two red lines are shown in the lower panel. **C** Comparison of the frequency of spontaneous calcium sparks and calcium waves. The parentheses indicate the number of cells where sparks or waves were detected out of a total of 38 myocytes examined.

Ca^2+^ sparks and waves were first detected at rest by two consecutive 10.24 s scan periods. Each 10.24 s scan was divided into 20 images (512×1024 pixels). Cells were then field stimulated (30 pulses at 0.5 Hz) to reach a steady-state SR calcium loading and Ca^2+^ transients were recorded in the line scan mode for another 2×10.24 s. Field stimulation was then stopped and Ca^2+^ sparks and waves were recorded again during two consecutive 10.24 s periods. Ca^2+^ sparks and waves were detected off-line as previously described for human atrial myocytes [4]. Briefly, Ca^2+^ sparks were detected as an increase in the signal mass of a 3 µm (25 pixels) section through the spark as shown in figure 1. For line scanning, sparks were accepted if the spark amplitude was at least twice the amplitude of the noise and the spark duration was between 25 and 300 ms. For each cell, the spark frequency before and after stimulation was determined and the spark frequency was normalized to the length of the cell scanned.

In a separate series of experiments, fluo-4 loaded myocytes were simultaneously subjected to perforated patch-clamp technique and fast confocal calcium imaging with a resonance scanning confocal microscope (Leica SP5 AOBS, Germany) in the frame-scanning mode. These experiments were performed using intra and extracellular solutions previously described [28]. The pipette solution contained (in mM): 109 aspartic acid, 47 CsCl, 3 Mg_2_ATP, 1 MgCl_2_, 5 Na_2_phosphocreatine, 0.42 Li_2_GTP, 10 HEPES and 250 µg/ml amphotericin B, (pH = 7.2). The extracellular medium contained (in mM): 127 NaCl, 5 TEA, 10 HEPES, 4 NaHCO_3_, 0.33 NaH_2_PO_4_, 10 glucose, 5 pyruvic acid, 2 CaCl_2_, 1.8 MgCl_2_, (pH = 7.4). Synchronization of confocal calcium imaging and recording of ionic currents was achieved using a Leica DAQ box and HEKA patch-master software. Patch-master was used to design electrophysiological protocols and to generate triggers for confocal image acquisition and event marking in the confocal time-lapse protocols created with the Leica Electrophysiology software module. Fluo-4 was excited at 488 nm with the laser power set to 20% of maximum and attenuation to 4%, and fluorescence emission was collected between 500 and 650 nm. Images of 140×512 pixels were recorded at a frame rate of 100 Hz. The width at half maximum (FWHM) and the duration of the calcium spark at half maximum (FDHM) as well as the peak amplitude (F/F_0_) was determined for each spark using a custom made spark detection program [29]. The average standard deviation of the noise in the fluorescent signal normalized to the baseline fluorescence (σ/F_0_) was 0.1, and only sparks with an amplitude larger than ΔF/F_0_ = 0.3 were accepted during the detection procedure. Moreover, the spark detection method employed is not based on an amplitude thresholding rule, but instead uses a multiscale wavelet detection that takes morphological features such as the width (more than 1 µm and less than 4.5 µm) and the characteristic decay time (less than 225 ms) of the calcium spark into account. This approach makes the detection method less prone to false alarms and provides a 10–30% increase in correctly detected sparks with respect to manual detection by an expert. Spontaneous release events that had a FWHM of more than 5 µm and a FDHM longer than 225 ms were counted as a Ca^2+^ wave when determining the frequency of Ca^2+^ waves.

### Data analysis

To minimize variations between myocytes isolated from the same animal, Ca^2+^ sparks recorded in the line-scanning mode were recorded in 6–8 cells from each animal and averaged. The average values were then used for statistical analysis unless otherwise stated. Values used for statistical analysis are expressed as mean±s.e.m. Two-tailed Student's t-test was used to assess significant differences when testing a specific effect. The threshold for statistical significance was p = 0.05. ANOVA was used for comparison of multiple effects and Student-Newman-Keuls post test was used to evaluate the significance of specific effects.

## Results

### Identification and characterization of calcium sparks in teleost cardiomyocytes

To determine whether spontaneous calcium release through the RyR2 occurs in the teleost heart, trout cardiomyocytes were used first because it is the best characterized teleost species. Myocytes were loaded with fluo-4 and local calcium release from the SR was detected using confocal line scanning. Figure 1A shows two consecutive confocal line-scan images from a trout atrial myocyte. Calcium sparks are indicated with arrowheads and the kinetics of the calcium sparks detected in a 3 µm wide band (indicated with red lines) are shown in the traces in below. For comparison, a calcium spark preceding a calcium transient elicited by electrical field stimulation is shown in figure 1B. Calcium sparks were observed in 16 of 38 atrial myocytes subjected to confocal line scanning, while preincubation with 30 µM of the SERCA inhibitor cyclopiazonic acid (CPA) abolished calcium sparks and calcium waves in myocytes from 4 different fish (data not shown). The average frequency of calcium sparks and calcium waves is summarized in figure 1C.

**Figure 2 pone-0023708-g002:**
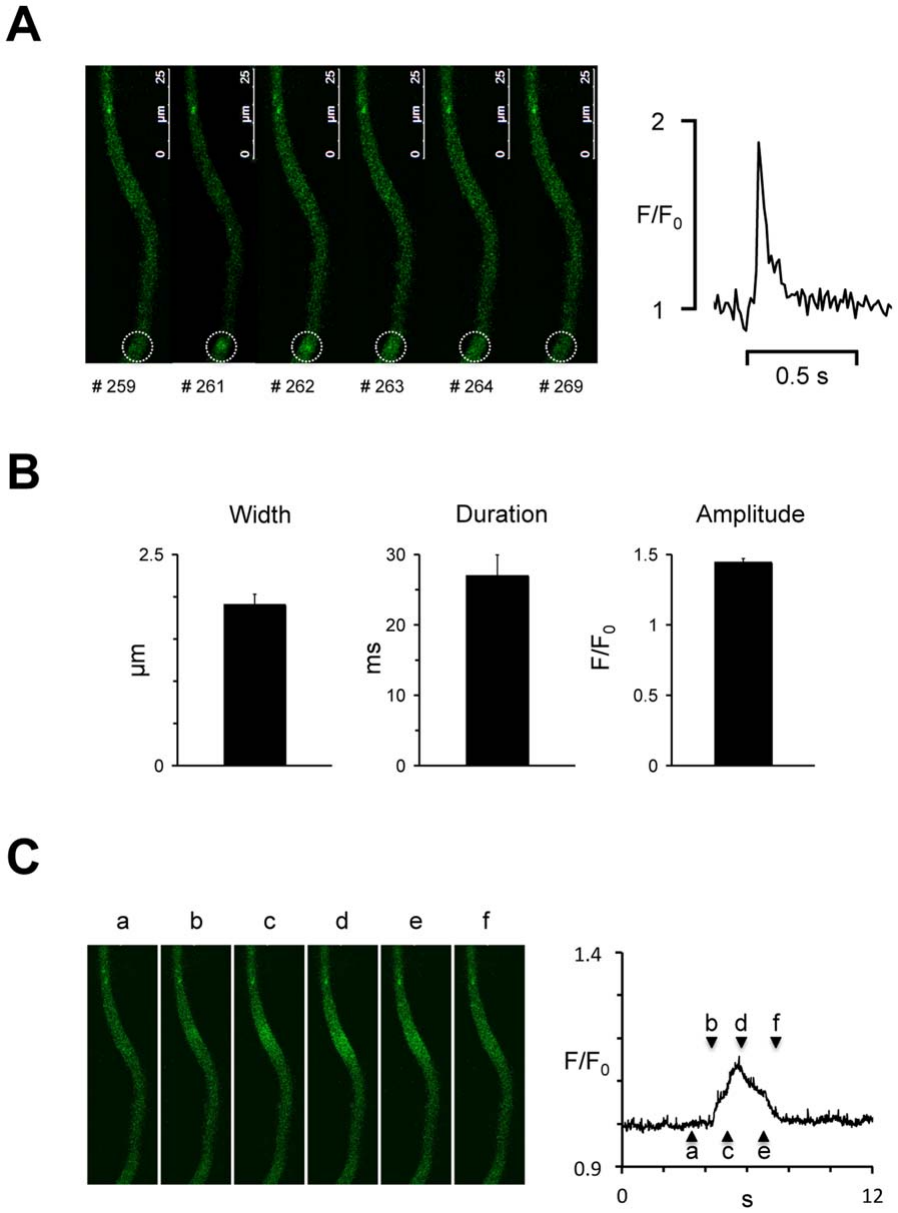
Calcium spark dimensions in trout atrial myocytes. **A** Consecutive images of a trout atrial myocyte recorded in the frame scanning mode at 100 Hz. The frame number is given below each image and the kinetics of the calcium spark detected in the white circle is shown on the right. **B** Average dimensions of 23 sparks from 8 trout atrial myocytes. **C** Consecutive images of the same trout atrial myocyte recorded during the propagation of a calcium mini-wave. The global calcium transient measured over the entire image field is shown on the right with letters indicating the temporal position of each image.

**Figure 3:**
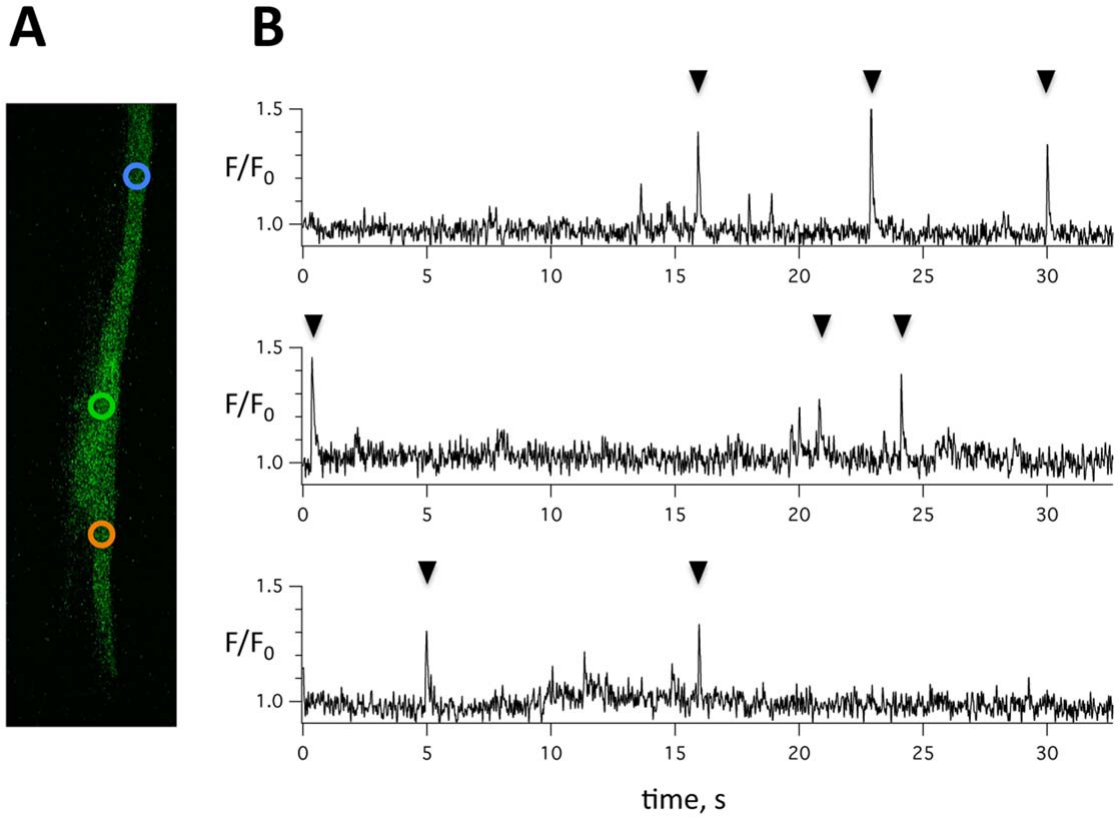
Calcium spark detection in zebrafish ventricular myocytes. **A** Zebrafish ventricular myocyte with indication of three spatially distinct spark sites. **B** Spark activity for each of the three spark sites in panel A. Values were normalized to the fluorescence intensity at baseline (F/F_0_) and sparks are indicated with arrowheads.

To further characterize the dimensions of calcium sparks in trout atrial myocytes the width (FWHM), the decay (FDHM), and the amplitude of calcium sparks were determined with the membrane potential clamped to −80 mV in myocytes simultaneously subjected to patch-clamp technique and fast confocal calcium imaging. Figure 2A shows consecutive two-dimensional images of a myocyte with a calcium spark. The corresponding calcium transient recorded in a 3 µm wide circle is shown on the right. Panel 2B summarizes the average FWHM, FDHM, and amplitude of 23 sparks recorded in 8 trout. The spark frequency was 6.1±1.4 min^−1^. Figure 2C shows consecutive images of the same myocyte during a calcium mini-wave with the resulting global calcium transient shown on the right. To test whether calcium sparks is a phenomenon particular to trout atrial myocytes, we also subjected trout and zebrafish ventricular myocytes to confocal calcium imaging, and sparks were detected with line-scanning in 6 of 15 trout ventricular myocytes (0.12±0.09 µm^−1^ min^−1^). Calcium sparks were also present in zebrafish ventricular myocytes. Figure 3A shows a zebrafish ventricular myocyte with indication of three spatially distinct spark sites, and the corresponding spark activity is shown for each spark site in figure 3B. Sparks were observed in (8/10) myocytes from 8 zebrafish, and the spark frequency varied from myocyte to myocyte (6.3±4.3 min^−1^, n = 8). The spark amplitude of 47 sparks from 8 zebrafish was 1.50±0.03, the spark width (FWHM) and duration (FDHM) at half maximum were 1.73±0.13 µm and 30.1±2.5 ms respectively.

### Role of the sarcoplasmic reticulum in teleost excitation-contraction coupling

**Figure 4:**
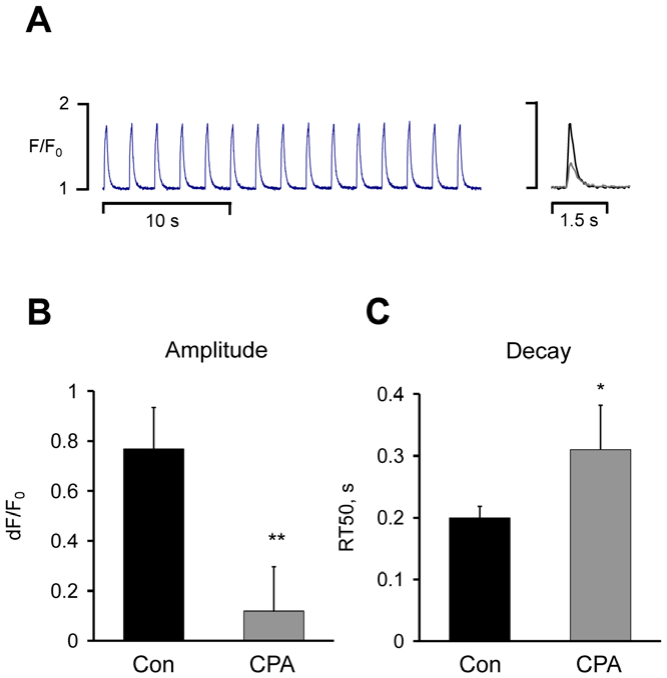
SERCA inhibition strongly reduces the calcium transient in trout myocytes. **A** Calcium transients recorded in a trout atrial myocyte subjected to repetitive stimulation before inhibition of SERCA with 30 µM cyclopiazonic acid (CPA). Calcium transients recorded before and after exposure to CPA are superimposed on the right. **B** Effect of CPA on the calcium transient amplitude. **C** Effect of CPA on the time to half maximal decay of the calcium transient. Significant effects of CPA are indicated with ** (p<0.01) and * (p<0.05).

The presence of calcium sparks in trout and zebrafish cardiomyocytes with characteristics similar to those reported in mammalian species strongly support a functional role of the ryanodine receptor in these species. To test this hypothesis, we compared field stimulated calcium transients in trout atrial myocytes before and after SR calcium depletion. Figure 4A shows consecutive calcium transients recorded before SR calcium depletion with 30 µM CPA and the right hand panel shows superimposed calcium transients recorded before and after exposure to CPA. Figure 4B shows that the amplitude of the calcium transient was dramatically reduced and the time constant for the decay of the calcium transient significantly increased in myocytes from four trout exposed to CPA for 40 minutes when compared to myocytes exposed to control solution for the same period of time.

### Calcium-dependent induction of afterdepolarizations in teleost cardiomyocytes

**Figure 5:**
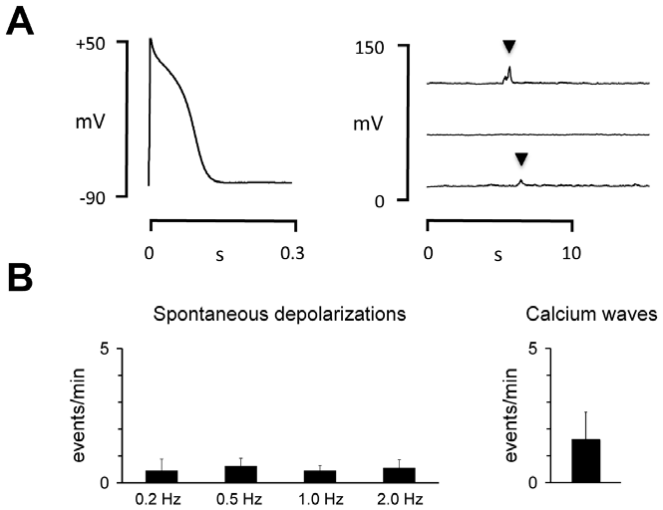
Spontaneous membrane depolarizations in trout atrial myocytes. **A** Recordings of action potential (left) and spontaneous membrane depolarizations (right) in a trout atrial myocyte subjected to current-clamp technique. Black arrowheads indicate spontaneous membrane depolarizations associated with a spontaneous cell shortening. **B** Dependency of the frequency of spontaneous membrane depolarizations on the preceding stimulation frequency (indicated below bars). For comparison the spontaneous calcium wave frequency at rest is shown in the right panel.

To further investigate the role of SR calcium release in trout cardiac myocytes, we used patch-clamp technique to detect afterdepolarizations resulting from spontaneous SR calcium release. Figure 5A shows representative recordings of an action potential (left panel) and three consecutive recordings at rest in an atrial myocyte. Notice two spontaneous membrane depolarizations in the top and bottom traces at rest. Figure 5B shows how the incidence of spontaneous membrane depolarizations depends on the stimulation frequency immediately prior to the recording of spontaneous membrane depolarizations (n = 9 myocytes). For comparison the frequency of spontaneous calcium waves recorded with confocal calcium imaging at −80 mV is shown on the right.

**Figure 6:**
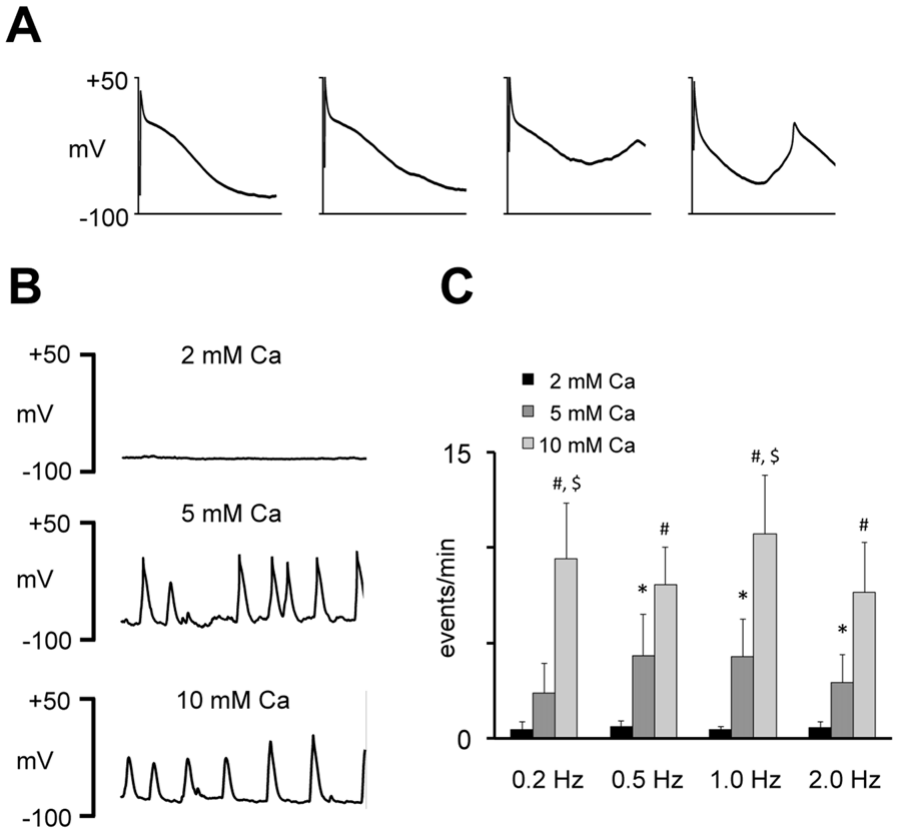
Extracellular calcium promotes early and delayed afterdepolarizations. **A** Triggered action potentials and spontaneous afterdepolarizations recorded in a trout atrial myocyte paced at 1 Hz in 5 mM extracellular calcium. Trace length is 200 ms. **B** Spontaneous membrane depolarizations recorded in a trout atrial myocyte exposed to increasing extracellular calcium concentrations (given above traces). Trace length is 10 s. **C** Effect of the extracellular calcium concentration on the frequency of spontaneous membrane depolarizations at rest. The pacing rate immediately before detection of the spontaneous membrane depolarizations is given below bars. ANOVA analysis showed that extracellular calcium significantly increased spontaneous membrane depolarizations. Significant differences are indicated with * (between 2 and 5 mM Ca^2+^); # (between 2 and 10 mM Ca^2+^); and $ (between 5 and 10 mM Ca^2+^).

In the mammalian heart it is generally accepted that spontaneous calcium release from the SR depends critically on SR calcium loading. Therefore, to test if increased SR calcium loading promotes spontaneous calcium release in trout, SR calcium loading was stimulated by increasing the extracellular calcium concentration from 2 to 5 or 10 mM calcium. As shown in figure 6, elevation of the extracellular calcium concentration induced both spontaneous afterdepolarizations in trout atrial myocytes submitted to repetitive stimulation (figure 6A) and spontaneous membrane depolarizations and action potentials at rest (figure 6B). Figure 6C summarizes the effect of increasing calcium concentrations on the frequency of spontaneous events recorded in 9 myocytes immediately after termination of stimulation at the pacing rate given below bars. A similar effect of the extracellular calcium concentration was observed in 15 trout ventricular myocytes. Spontaneous membrane depolarizations were observed in 7/15 myocytes and elevation of the extracellular [Ca^2+^] increased their frequency from 0.27±0.18 to 1.06±0.33 events/min (p<0.05).

**Figure 7:**
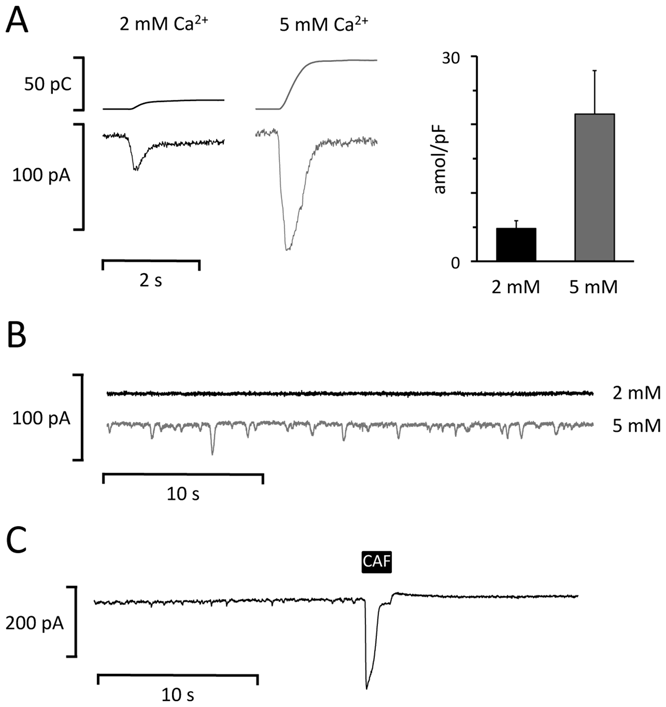
Calcium dependency of spontaneous calcium release and SR calcium load. **A** Transient inward Na^+^-Ca^2+^ exchange current elicited by a rapid caffeine application (lower trace) and its time integral (upper trace). The extracellular [Ca^2+^] is given above traces. The calcium loading (estimated from the time integrals) with 2 and 5 mM extracellular [Ca^2+^] is shown on the right. **B** Whole membrane currents recorded in a trout atrial myocyte at -80 mV with 2 mM (black trace) and 5 mM (grey trace) extracellular [Ca^2+^]. Traces are displaced by 50 mV for clarity. Notice the activation of numerous spontaneous transient inward currents in 5 mM Ca^2+^. **C** Whole membrane current recorded at −80 mV with 5 mM extracellular [Ca^2+^] before, during, and after transient application of 10 mM caffeine (CAF). Notice that the spontaneous inward currents disappear after CAF.

To verify that calcium is spontaneously released from the SR and dependent on the SR calcium loading, we used rapid caffeine applications to clear the SR calcium content and to estimate the SR calcium. First, we determined the effect of increasing the extracellular [Ca^2+^] from 2 to 5 mM on SR calcium loading by integrating the transient inward Na^+^-Ca^2+^ exchange current elicited by rapid caffeine application. Figure 7A shows the transient inward Na^+^-Ca^2+^ exchange current elicited by a rapid caffeine application (lower trace) and its time integral (upper trace) recorded with 2 and 5 mM extracellular [Ca^2+^]. The right panel shows the average SR calcium load with 2 and 5 mM extracellular [Ca^2+^] in trout atrial myocytes (n = 5). Similar results were obtained in trout ventricular myocytes (n = 4), and table 1 summarizes the effects of the extracellular [Ca^2+^] on the frequency of spontaneous membrane depolarizations and SR calcium loading in trout atrial and ventricular myocytes. Figure 7B shows that increasing the extracellular [Ca^2+^] from 2 to 5 mM concurrently induced spontaneous transient inward currents that were associated with cell contractions (not shown). Figure 7C shows that these transient inward currents were abolished when the SR calcium content was cleared with a brief caffeine pulse.

**Table 1:** Effect of the extracellular [Ca^2+^] on the frequency of spontaneous membrane depolarizations and the SR calcium load in trout atrial and ventricular myocytes.

	Spontaneous depolarizations				SR calcium loading			
[Ca^2+^]		2 mM Ca	5 mM Ca	10 mM Ca		2 mM Ca	5 mM Ca	10 mM Ca
	n	events/min	events/min	events/min	n	amol/pF	amol/pF	amol/pF
**Atrium**	9	0.61±0.30^χ^	4.33±2.19*^,χ^	8.07±1.99*^,χ^	5	4.8±1.1	21.5±6.3*	
**Ventricle**	15	0.27±0.18	1.06±0.33*	1.07±0.36*	4	5.0±0.6		15.0±0.4*

Values significantly different from the corresponding value in 2 mM Ca^2+^ are indicated with *. A significant difference between trout atrial and ventricular myocytes is indicated with χ.

## Discussion

### Role of the sarcoplasmic reticulum in teleost cardiomyocytes

The present study provides direct evidence for an active intervention of the SR in calcium handling on a beat-to-beat basis in trout. Thus, depletion of the SR calcium content strongly reduced the intracellular calcium transient (by 81%), a value that is comparable to those reported in mammalian cardiomyocytes [1]. However, the time course of inhibition of SR calcium uptake was slower than that observed in mammalian ventricular myocytes [30], [31]. This may at least partly be due to the 3–4 fold higher SR calcium load at steady state [23], [25] and a smaller fractional calcium release from the SR in trout atrial myocytes [15], [24], [32]. As a result, it will take longer time from the onset of SERCA inhibition until the SR is depleted. In agreement with this it also takes a large number of stimulation pulses to fully reload the SR after calcium depletion with caffeine [25], [33].

The present results support previous indirect findings showing that the SR can contribute to the activation of contraction in trout ventricle [34]. It also agrees with a large SR calcium storage capacity in trout cardiomyocytes [23], [25], [32], [33], and SR calcium uptake-rates sufficiently high to allow SR calcium re-uptake on a beat-to-beat basis at physiological stimulation frequencies and temperatures [23], [32]. Furthermore, electrophysiological protocols designed to trigger and measure SR calcium release estimated it be near 50% of the total calcium transient [15], [24], [32]. The results presented here suggest that the contribution could be even larger in trout atrial myocytes.

### Spontaneous sarcoplasmic reticulum calcium release in teleost cardiomyocytes

The above findings strongly suggest that the SR does intervene in the excitation-contraction coupling in trout cardiomyocytes. In addition to this, the present study provides for the first time direct measurements of both spontaneous and triggered calcium release from the SR in trout and zebrafish, showing that the basic features of calcium sparks such as dimensions and duration are similar to those of calcium sparks recorded in mammalian and human cardiomyocytes under comparable conditions [4], [35]. Importantly, the frequency of calcium sparks and spontaneous calcium waves in trout atrial myocytes are also within the range of values previously reported in mammalian and human atrial myocytes under similar experimental conditions [4], [35], [36], supporting the notion that the key features of SR calcium release through the ryanodine receptor have been conserved phylogenetically. Interestingly, calcium sparks with similar properties were also observed in zebrafish ventricular myocytes. Since the L-type calcium current density is large in zebrafish [27], [37], this finding shows that myocytes can have a high SR activity even though the I_Ca_ density is sufficiently large to provide for the calcium required for the activation of contraction.

It is not clear why a previous study on isolated trout cardiomyocytes failed to detect calcium sparks under physiological conditions [22], but initial experiments of the present study showed that trout cardimoycytes are highly sensitive to excess laser intensity.

Our recordings of spontaneous membrane depolarizations induced by calcium release from the SR show that spontaneous SR calcium release can even produce afterdepolarizations in trout atrial myocytes, and in agreement with the effect of the extracellular calcium concentration on spontaneous calcium release reported in mammalian cells [38] we here observe that elevation of the extracellular calcium concentration dramatically increases the frequency of spontaneous membrane depolarizations and spontaneous action potentials in both trout atrial and ventricular myocytes.

### The teleost heart as a model to study SR function and cardiac arrhythmogenesis

The growing interest in teleost species such as medaka and zebrafish as vertebrate models for studies of cardiovascular disease and regeneration highlights the importance and broader physiological relevance of the present findings. So far, the teleost cardiomyocyte model has been considered of limited translational value because a minor or rudimentary role of the SR in excitation-contraction coupling of the ectothermic vertebrate heart has been the prevailing paradigm. However, the present work demonstrates that the SR is functionally well developed in teleost cardiomyoctes and shares fundamental features such as calcium sparks (figures 1–3) and the ability to trigger calcium induced afterdepolarizations (figures 5–6) with the human heart [4], [39], [40]. Particularly, SR function in neonatal mammalian and adult teleost cardiomyocytes appears to have many common features. Morphologically, both are slim and elongated and lack t-tubules [20], [41], [42]. The SR also has about 3-fold higher calcium storing capacity in neonatal than adult rabbit ventricular myocytes [43], and reverse mode Na^+^-Ca^2+^ exchange has been shown to play a prominent role in both trout [15], [16] and neonatal cardiomyocytes [44], [45].

This, combined with a strong similarity between teleost and human action potential morphology [26], [37], [46] (as opposed to the short and triangular shaped action potentials of murine cardiomyocytes), the robustness of isolated teleost cardiomyocytes, and the availability of morpholino antisense oligonucleotides in zebrafish, proposes the teleost heart as a relevant model to study genetic impact on SR function and cardiac arrhythmogenesis.

## Conclusions

In summary, this is to the best of our knowledge the first report demonstrating the presence of robust calcium sparks in the teleost heart. Moreover, the dimensions, duration and frequency of calcium sparks in trout and zebrafish cardiomyocytes are comparable to values reported in mammalian and human cardiomyocytes, suggesting that the basic features of calcium release through the ryanodine receptor are conserved from teleost to mammal. Moreover, the slow but dramatic reduction of the calcium transient upon inhibition of SR calcium uptake suggests that the SR plays a prominent role in calcium handling on a beat-to-beat basis in trout and zebrafish cardiomyocytes, proposing them as a new model useful for studying the impact of genetic or pharmacological manipulation of SR function on cardiac arrhythmogenesis.
